# Screening high-risk clusters for developing birth defects in mothers in Shanxi Province, China: application of latent class cluster analysis

**DOI:** 10.1186/s12884-015-0783-x

**Published:** 2015-12-22

**Authors:** Hongyan Cao, Xiaoyuan Wei, Xingping Guo, Chunying Song, Yanhong Luo, Yuehua Cui, Xianming Hu, Yanbo Zhang

**Affiliations:** Division of Health Statistics, School of Public Health, Shanxi Medical University, No. 56 South Xinjian Road, Taiyuan, Shanxi 030001 PR China; Population and Family planning Commission of Shanxi province, No. 11 North Beiyuan Road, Taiyuan, Shanxi 030006 PR China; Department of Statistics and Probability, Michigan State University, East Lansing, MI 48824 USA; Department of Developmental Pediatrics, Affiliated Children’s Hospital of Shanxi Medical University, No. 15 North Xinmin Road, Taiyuan, Shanxi 030013 PR China

**Keywords:** Maternal exposure, Clusters, Birth defects, China, Latent class cluster analysis

## Abstract

**Background:**

Few studies on cluster-based synthetic effects of multiple risk factors for birth defects have been reported. The present study aimed to identify maternal exposure clusters, explore the association between clusters of risk factors and birth defects, and further screen women with high risk for birth defects among expectant mothers.

**Methods:**

Data were drawn from a large-scale, retrospective epidemiological survey of birth defects from 2006 to 2008 in six counties of Shanxi Province, China, using a three-level stratified random cluster sampling technique. Overall risk factors were extracted using eight synthetic variables summed and examined as a total risk factor score: maternal delivery age, genetic factors, medical history, nutrition and folic acid deficiency, maternal illness in pregnancy, drug use in pregnancy, environmental risk factors in pregnancy, and unhealthy maternal lifestyle in pregnancy. Latent class cluster analysis was used to identify maternal exposure clusters based on these synthetic variables. Adjusted odds ratios (AOR) were used to explore associations between clusters and birth defects, after adjusting for confounding variables using logistic regression.

**Results:**

Three latent maternal exposure clusters were identified: a high-risk (6.15 %), a moderate-risk (22.39 %), and a low-risk (71.46 %) cluster. The prevalence of birth defects was 14.08 %, 0.85 %, and 0.52 % for the high-, middle- and low-risk clusters respectively. After adjusting for maternal demographic variables, women in the high-risk cluster were nearly 31 times (AOR: 30.61, 95 % CI: [24.87, 37.67]) more likely to have an infant with birth defects than low-risk women.

**Conclusions:**

A high-risk group of mothers in an area with a high risk for birth defects were screened in our study. Targeted interventions should be conducted with women of reproductive age to improve neonatal birth outcomes in areas with a high risk of birth defects.

## Background

Birth defects are defined as any abnormality in functions, structures, and metabolism of the fetus that is developed in the maternal uterus [1]. Birth defects are a leading cause of infant death, accounting for 21 % of all infant deaths [2]. Having a child with birth defects can create irreversible damage to families and to society as a whole. Shanxi Province, the largest coal mining base in China, has reported a rate of birth defects of 8.39 % in recent studies [3], a much higher rate than the average prevalence of birth defects in China (5.6 %) and the US (3 %) [4, 5]. Therefore, identification of maternal exposure risk factors before and during pregnancy, as well as screening high-risk groups of women of reproductive age in high-risk areas is important for early intervention for birth defects.

To date, there have been many epidemiologic approaches to examine the impact of single risk factors, adjusting for confounding variables using generalized linear models (e.g., logistic regression or Poisson regression), such as folic acid supplementation, maternal delivery age, or alcohol consumption [1, 6, 7]. However, birth defects are caused by the complex synergistic effects of multiple, interrelated risk factors, including genetic factors, unhealthy lifestyles during pregnancy, and other maternal exposures [1, 8].

Unlike the traditional single factors approaches, latent class cluster analysis (LCCA) is a model-base clustering approach that examines the interrelationships among multiple risk factors and classifies similar objects into groups [9, 10]. Previous research has extracted clusters from a limited number of maternal factors to assess synthetic effects using LCCA, for example, assessing the relationship between dietary patterns (based on series of foods) and birth defects [11]. However, research is still limited to some dietary factors, and no method of expressing the distribution of populations at high-risk of developing birth defects has been established. There is a discrepancy between synthetic effects based on overall maternal exposures and birth defects. To address this discrepancy, it is essential to examine the synthetic effects of all possible risk factors for birth defects identified in previous research, and to screen high-risk groups of expectant mothers who live in high-risk areas using new statistical methods.

In 2009, we conducted a large-scale, retrospective epidemiological survey to assess the prevalence of and likely risk factors for birth defects from 2006 to 2008 in six counties in Shanxi Province, China. Based on these data, the aims of our present study were twofold: 1) to extract different latent maternal exposure clusters based on overall risk factors in the high-risk area using LCCA; and 2) to explore the association between latent clusters and birth defects, screening expectant mothers developing birth defects in high-risk clusters. Identifying individuals in risk clusters characterized by different risk factors will be useful to inform targeted interventions for improving neonatal birth outcomes in areas with a high risk of birth defects.

## Methods

### Study design and participants

A large-scale, retrospective epidemiological survey of birth defects was conducted in six counties in Shanxi Province, China (Pingding, Dai, Fenyang, Huairen, Zhongyang, and Jiaokou). The survey was conducted by Population and Family Planning Commission (PFPC) of Shanxi Province, using a three-level stratified random cluster sampling technique [12–14]. Three different economic levels were identified for counties in Shanxi Province, and defined as the third stratum. Two counties were randomly selected from each economic level (developed counties: Huairen and Fenyang; developing counties: Dai and Jiaokou; underdeveloped counties: Zhongyang and Pingding), and then one township was randomly selected from each of the six counties. Data for all live infants and their mothers for 2006–2008 were obtained from each township. Data for 36,716 live infants and their mothers (abnormal: 524; normal: 36,192) in Shanxi Province were obtained.

Mothers were interviewed face-to-face by trained, local investigators going from house to house. Questionnaires designed by PFPC of Shanxi Province were completed by investigators during the interviews. The investigation team in each town comprised 10–12 members (including at least two clinical doctors), with two town PFPC workers acting as supervisors. Diagnostic information for birth defects was obtained from previous medical records. Unclear birth outcomes were examined by clinical doctors.

During the survey, all completed questionnaires were monitored and checked by supervisors and investigators on the same day. When errors and/or missing values were detected, those mothers were re-interviewed. Additionally, 1 % of mothers were reviewed randomly by PFPC of Shanxi Province workers via a telephone interview. Informed consent was obtained from each mother at the beginning of the survey. The Human Research Ethics Committee of Shanxi PFPC reviewed and approved the epidemiological survey and our in-depth following up analysis.

### Data collection

The questionnaire was divided into six sections. The first section recorded mothers’ demographic data including age, residence (urban/rural), education, occupation, and annual net income per capita. The second section concerned family history (parental consanguinity, birth defects in previous infants and immediate family members). The third section consisted of a series of Yes/No questions about maternal medical history such as hepatitis, epilepsy, and diabetes. The fourth section covered premarital and pre-conception health guidance. The fifth section investigated maternal exposures during the first 12 weeks of pregnancy, and consisted of six parts, each with a specific series of questions: meat and vegetable consumption, folic acid supplementation, maternal illness in pregnancy, drug use in pregnancy, environmental risk factors in pregnancy and unhealthy maternal lifestyle in pregnancy. Response options for meat and vegetable consumption were: 0, 1–2, or ≥ 3 times per week. Folic acid supplementation had a Yes/No option (“Yes” was selected if supplementation had lasted for at least 3 months within the 3 months before or after the start of pregnancy). Maternal illness in pregnancy and drug use in pregnancy were measured with a series of Yes/No questions. Environmental risk factors in pregnancy and unhealthy maternal lifestyle in pregnancy were measured with a series of questions with response options of never (0), occasionally, or often, with the exception of computer use (0, < 20 h, 20–40 h, >40 h), and pollution source in area of residence (Yes/No, such as coal mines, coal-fired power plants, chemical plants). The final section collected demographic data for offspring. If an infant with birth defects was present, further questions were asked on diagnostic methods and types of birth defects.

### Statistical analysis

Statistical analyses were carried out in a three-step process. First, we extracted eight important indicator variables: maternal delivery age, genetic factors, medical history, nutrition and folic acid deficiency, maternal illness in pregnancy, drug use in pregnancy, environmental risk factors in pregnancy, and unhealthy maternal lifestyle in pregnancy. Each indicator variable included multi-risk factor items except for maternal delivery age, and were summed and examined as a “total risk factor score” for data dimensionality reduction: ranging from 0 to N (the number of risk factors in each indicator variable) [15]. The risk factors in each indicator are described in Table 1. All eight indicators were regarded as continuous variables and standardized for LCCA.

**Table 1:** Description of eight indicator variables in latent class cluster analysis to identify latent clusters^a^

Indicator variable	Risk factors^b^	Min^c^	Max^c^
Maternal delivery age	Maternal delivery age	14	55
Genetic factors	Parental consanguinity	0	3
	Birth defects in immediate family members		
	Birth defects in previous infants		
Medical history	Hepatitis	0	6
	Anemia		
	Epilepsy		
	Heart disease		
	Diabetes		
	Thyroid disease		
	Spontaneous abortion		
	Other		
Nutrition and folic acid	Meat deficiency	0	3
	Vegetable deficiency		
	Folic acid deficiency		
Maternal illness	Fever	0	6
	Cold		
	Threatened abortion		
	Reproductive tract infections		
	Hyperemesis gravidarum		
	Rash and fever		
	Other		
Drug use	Cold medicines	0	7
	Antiemetic		
	Antibiotic		
	Antiepileptic		
	Sedative		
	Contraceptive		
	Abortion prevention agent		
	Other		
Environmental risk factors	Pesticides	0	6
	Chemical fertilizers		
	X-rays		
	Computer use		
	Pets		
	Pollution source in area of residence		
Unhealthy lifestyle	Periconceptional smoking	0	4
	Family member smoking		
	Periconceptional drinking		
	Family member drinking		

^a^Series of risk factors were summed as a total risk factor score for each indicator variable

^b^All risk factors were transformed into 0/1 variables: 0 = No, 1 = Yes

^c^Min-max values for each indicator variable

Second, LCCA was performed with the eight indicator variables using LatentGOLD 4.5 [16]. LCCA [9, 17, 18] is a method that involves continuous and classified indicators, based on local independence, which assumes that the items are independent within each latent class, and concentrates on data reduction and classifying the population into different latent classes or subgroups. LCCA for categorical indicators assuming multinomial distributions are called latent class analysis, whereas latent profile analysis is designed for continuous variables. This study was undertaken on continuous indicators, assuming multivariate normal distribution within latent classes with parameters *μ*_*k*_ and ∑_*k*_ in general; therefore an LCCA model for *p* manifest variables (*j* = 1, . . ., *p*) with *K* classes (*k* = 1, . . ., *K*) is stated as:

$$ f\left({y}_i\right)={\displaystyle \sum_{k=1}^K{\pi}_k{f}_k\left({y}_i\Big|{\mu}_k,{\sum}_k\right)} $$

$$ ={\displaystyle \sum_{k=1}^K{\pi}_k{\displaystyle \prod_{j=1}^p\frac{1}{\sqrt{2\pi {\sigma}_{jk}^2}}}} \exp \left(\frac{-{\left({y}_{ij}-{\mu}_{jk}\right)}^2}{\sigma_{jk}^2}\right), $$

where *y*_*i*_ is an object’s score on a set of manifest variables, *π*_*k*_ is the marginal probability of latent class *k*, and *μ*_*jk*_ and *σ*_*jk*_^2^ are the mean and variance for manifest variables *j* in class *k*, respectively. We estimated means and relative frequencies of classes, which were different from the probabilities of class-specific responses. The posterior probability of assigning respondents to the *k* class, by Bayes’ theorem, is equal to:

$$ P\left(k\Big|{y}_i\right)=\frac{\pi_k{f}_k\left({y}_i\Big|{\mu}_k,{\sum}_k\right)}{{\displaystyle \sum_{k=1}^K{\pi}_k{f}_k\left({y}_i\Big|{\mu}_k,{\sum}_k\right)}} $$

Finally, individuals were classified into different latent classes. Once ≥1-class models were obtained in the exploratory LCCA, goodness-of-fit indicators such as Akaike information criterion (AIC), consistent Akaike information criterion (CAIC), Bayesian information criterion (BIC), and entropy and classification errors were used to determine the model of best fit. In general, smaller AIC, CAIC, BIC and classification errors indicate a better model, whereas the opposite applies for the entropy.

Controlling for residence, education, and annual net income per capita, we conducted multivariate logistic regression to explore the association between exposure clusters and birth defects using SPSS Version 17.0 (SPSS Statistics for Windows, Version 17.0. Chicago: SPSS Inc.).

## Results

### Maternal demographic characteristics

The average maternal delivery age was 26.3 years. More mothers were rural residents (53.56 %), than urban residents (46.44 %). Most mothers had junior high school education (72.87 %); 1.02 % had no schooling; 14.74 % had elementary school education; 8.66 % had senior high school or technical secondary school education; and, 2.70 % had junior college education or above. Around 11.0 % of mothers had an annual net income per capita of less than 1000 Chinese Yuan (¥); 26.32 % had an annual income of 1000–2000¥; 20.22 % of 4000–8000¥; and, 7.51 % had an annual income of more than 8000¥.

### Latent class cluster analysis

We conducted 2- to 5-cluster models. Table 2 presents the goodness-of-fit measures for the four models assessed. The BIC, AIC and CAIC values reduced sharply from the 2-cluster model to the 3-cluster model, and then decreased slowly after the 3- cluster model. Furthermore, the entropy in the 3-cluster model was 1, and the error was 0; therefore, the 3-cluster model was chosen as the final model.

**Table 2:** Goodness-of-fit measures of the four different class models

Model	LL^a^	BIC	AIC	CAIC	Entropy	Errors^b^	Npar^c^
2-cluster	116915.17	−233483.48	−233764.34	−233450.48	0.9997	0.0000	33
3-cluster	280772.34	−561019.12	−561444.67	−560969.12	1.0000	0.0000	50
4-cluster	331698.74	−662693.25	−663263.49	−662626.25	1.0000	0.0000	67
5-cluster	396908.65	−792934.38	−793649.30	−792850.38	0.9981	0.0007	84

^a^LL = Log likelihood

^b^Error = Classification errors

^c^Npar = Number of parameters estimated

**Fig. 1:**
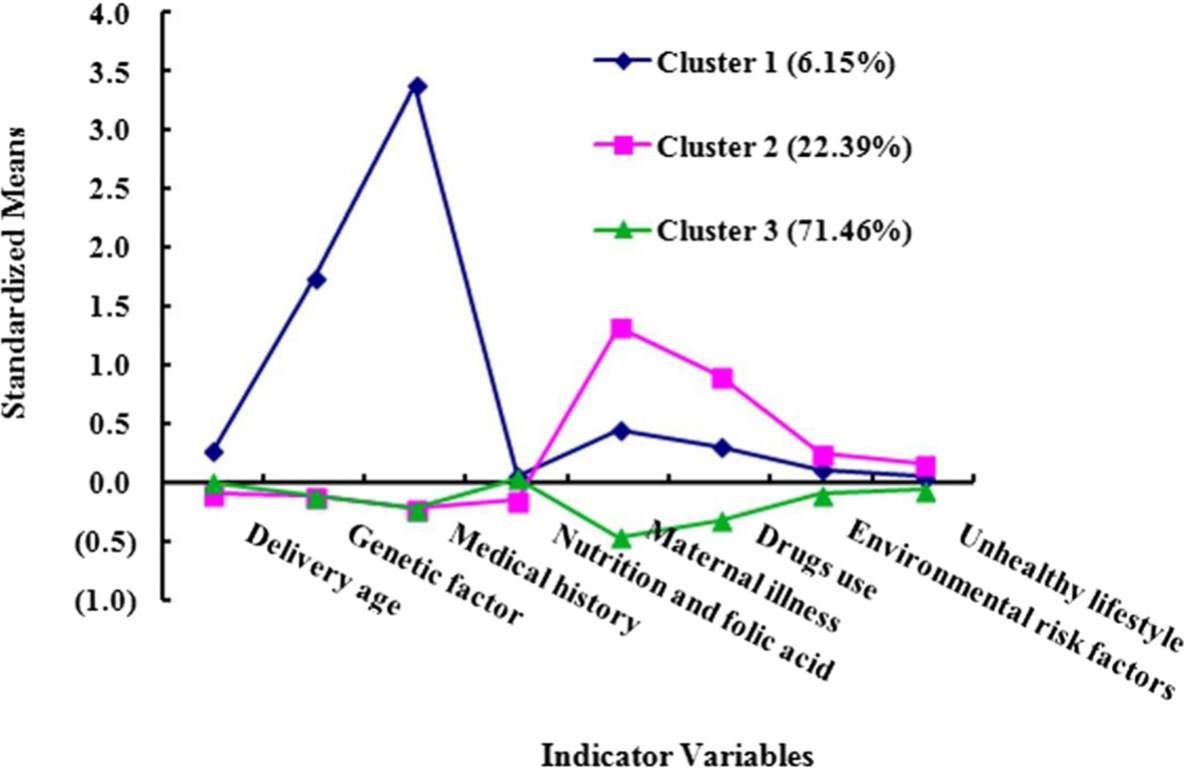
Standardized means of three clusters across the eight indicator variables. Legend: Cluster 1, 2, and 3 refer to the high-risk, moderate-risk and low-risk cluster, respectively

Table 3 presents the means, standard deviations and the multiple comparisons across the eight indicator variables for the three derived latent classes. Cluster 1 was the smallest group (6.15 %, *n* = 2258), but consisted of the highest levels of maternal delivery age, genetic factors, medical history, nutrition and folic acid deficiency, and comparatively high levels of the other four indicator variables. Therefore, Cluster 1 was characterized as the high-risk cluster. Cluster 2 (22.39 %, *n* = 8221) comprised the highest level of illness in pregnancy, drug use in pregnancy, environmental risk factors in pregnancy, unhealthy lifestyle in pregnancy, and the lowest levels of the other four indicator variables, and was considered to be the moderate-risk cluster. Cluster 3 was the largest group (71.46 %, *n* = 26,237), characterized by the lowest levels of indicator variables except for a comparatively high level of nutrition and folic acid deficiency and a moderate level of high maternal delivery age, and was considered the low-risk cluster. Figure 1 describes the standardized means of the three clusters across each indicator variable.

**Table 3:** Standardized characteristics of latent classes among respondents^a^

Indicator variables	Cluster 1^a^	Cluster 2^a^	Cluster 3^a^	*F* ^b^	Multiple comparisons^c^
1 Maternal delivery age	**0.27 (1.07)**	−0.09 (0.97)	0.00 (1.00)	113.39^*^	1 > 3 > 2
2 Genetic factors	**1.73 (3.61)**	−0.11 (0.00)	−0.11 (0.00)	4498.98^*^	1 > 2, 1 > 3
3 Medical history	**3.37 (2.03)**	−0.22 (0.00)	−0.22 (0.00)	53915.62^*^	1 > 2, 1 > 3
4 Nutrition and folic acid	**0.05 (1.02)**	−0.14 (0.99)	**0.04 (1.00)**	100.06^*^	1 > 2, 3 > 2
5 Maternal illness	0.45 (1.46)	**1.33 (1.18)**	−0.46 (0.00)	23225.03^*^	2 > 1 > 3
6 Drug use	0.31 (1.40)	**0.91 (1.66)**	−0.31 (0.00)	6468.21^*^	2 > 1 > 3
7 Environmental^e^	0.13 (1.11)	**0.27 (1.15)**	−0.10 (0.92)	445.78^*^	2 > 1 > 3
8 Unhealthy lifestyle	0.05 (1.00)	**0.17 (0.98)**	−0.06 (1.00)	160.82^*^	2 > 1 > 3

^a^Entries in column = Mean (Standard deviation)

^b^One-way ANOVA was conducted to compare means of each indicator across three clusters, ^*^ *P* <0.0001

^c^Student–Newman–Keuls test was used for multiple comparisons among different clusters. Means were significantly different at *P* < 0.05

^d^Bold text indicated highest mean of the indicator in three different latent classes

^e^Environmental = Environmental risk factors

Further, each participant was assigned to the most likely class based on the known parameters of the three-cluster latent structure. Table 4 describes the recruitment probabilities and LCCA cluster assignment for respondents.

**Table 4:** Class assignment for part of respondents^a^

Y1	Y2	Y3	Y4	Y5	Y6	Y7	Y8	*P*(1|*y*)	*P*(2|*y*)	*P*(3|*y*)	Cluster
0.77	7.28	−0.22	−1.15	1.29	1.66	1.73	0.74	1	0	0	1
−0.87	−0.11	4.04	−1.15	−0.46	−0.31	0.56	0.74	1	0	0	1
0.56	7.28	−0.22	0.50	1.29	3.63	1.73	0.74	1	0	0	1
−0.05	−0.11	−0.22	0.50	−0.46	−0.31	0.56	0.74	0	0	1	3
−0.05	7.28	−0.22	0.50	1.29	3.63	0.56	−0.38	1	0	0	1
−0.05	−0.11	−0.22	0.50	−0.46	−0.31	1.73	0.74	0	0	1	3
−0.67	14.68	−0.22	0.50	4.79	3.63	0.56	0.74	1	0	0	1

^a^Y1–Y8 represent standardized values of the eight indicator variables. P(1|*y*), P(2|*y*) and P(3|*y*) denote the probability of belonging to a class, given *y*. Cluster denotes for class assignment

### Association of birth defects with identified latent classes

We explored the association between birth defects and the identified latent classes. Table 5 shows the prevalence of birth defects for the three maternal exposure factor clusters. The high-risk cluster comprised the fewest individuals, but had the highest prevalence of birth defects (14.08 %). Those in the moderate-risk cluster had a prevalence of birth defects of 0.85 %, which was higher than that of the low-risk cluster (0.52 %). Additionally, adjusting for residence, education, and annual net income per capita, the associations between birth defects and each of the three latent classes were examined using logistic regression, with the low-risk cluster serving as the reference (Table 6). Mothers in the moderate-risk cluster were more likely (AOR: 1.64, 95 % CI: [1.22, 2.19]) to have infants with birth defects than those in the low-risk cluster. The strength of this association was significantly greater for mothers in the high-risk cluster, with these mothers being nearly 31 times more likely than those in the low-risk cluster to have infants with birth defects (AOR: 30.61, 95 % CI: [24.87, 37.67]). Meanwhile, this study showed that annual net income per capita was significantly associated with birth defects (AOR: 0.77, 95 % CI: [0.71, 0.84]).

**Table 5:** Comparisons of the prevalence of birth defects in the three risk clusters

Clusters	N	Prevalence (%)	*χ* ^2^	Multiple comparisons^a^
High-risk cluster	2259	318 (14.08)	2742.94	1 > 2 > 3
Moderate-risk cluster	8219	70 (0.85)		
Low-risk cluster	26238	136 (0.52)		
Total	36716	524 (1.43)		

^a^ *P* values were adjusted using Bonferroni method for multiple comparisons

**Table 6:** Associations between birth defects and risk clusters^a^

Variables	Coefficient (S.E.)	AOR	95 % CI
Residence	−0.03 (0.10)	0.98	(0.81–1.18)
Education	0.01 (0.08)	1.01	(0.87–1.17)
Income	−0.26 (0.04)	0.77^*^	(0.71–0.84)
Moderate-risk cluster	0.49 (0.15)	1.64^*^	(1.22–2.19)
High-risk cluster	3.42 (0.11)	30.61^*^	(24.87–37.67)

^a^Associations between birth defects and risk clusters were assessed by logistic regression adjusting for residence, education and annual net income per capita. Low-risk cluster was the referent

^*^Denote *P* < 0.0001

## Discussion

Birth defects with serious outcomes and complex maternal exposures are highly prevalent in Shanxi Province. The results of the present study showed that a synthetic effect of overall maternal exposures occurred in areas with a high-risk of birth defects. After extracting three maternal risk clusters using LCCA, mothers were classified into three independent clusters (a high-risk cluster, a moderate-risk cluster, and a low-risk cluster). Each cluster differed in maternal exposures and had a different risk of having an infant with birth defects. The high-risk cluster consisted of the smallest proportion of the study population (6.15 %); however, it had the highest birth prevalence of birth defects at approximately 14.08 %. Furthermore, after adjusting for maternal demographic variables, we found that women in the high-risk cluster were nearly 31 times more likely to have offspring with birth defects compared with the low-risk cluster. This suggests that future government-led, integrated interventions in areas with a high-risk of birth defects may be necessary.

Our findings confirmed a significant negative effect associated with all risk factors, and extended the results of single-explanatory risk studies based on a generalized linear model adjusted for confounding variables. The findings in this study also serve as an extension to the results of previous latent class studies based on special dietary factors [11]. Research on integrating multiple risk factors and classifying a population into subgroups for targeted interventions using a cluster-based approach have received much attention in other fields, including psychiatry [19, 20], lifestyle behaviors with education [21], truant youth profiles [22], and multiple risk factors in primary care [10]. However, to date, there is no published research that has examined overall maternal exposures by identifying a total risk factor score for each indicator factor, and used LCCA to explore the synergistic effects of multiple risk factors and to screen high-risk groups in a high-risk birth defects area.

The high-risk cluster had the highest prevalence of birth defects, and comprised of mothers with high values in maternal delivery age, genetic factors, medical history, and nutrition and folic acid deficiency. Therefore, those in a high-risk cluster should receive the most attention in terms of implementing interventions targeting behavioral change. Our study found that the rate of birth defects is higher in older maternal age group (≥35 years) (2.02 %) than those in younger maternal age group (<35) (1.40 %) (*χ*^2^ = 7.03, *p*-value < 0.05). This result is consistent with a research finding conducted earlier in Shanxi Province [23]. Delivering at younger reproductive age can help to improve infant outcomes and reduce the occurrence of birth defects [7, 24]. In addition, further interventions should be focused on avoiding the dangers of inbreeding and reducing potential risk due to medical and familial inheritance history. Meanwhile, 39.60 % of pregnant women in our study reported that they took folic acid, much more than a previously reported study in Shanxi during 2003 (9.38 %) [25]. Even though the percentage of women taking folic acid was significantly increased over the years as supported by National Birth Defects Prevention Program [26], there is still a large need to increase this proportion as evidenced by the high-risk cluster with nutrition and folic acid deficiency in our study.

The moderate-risk cluster (22.39 %) had a prevalence of birth defects of 0.85 %. This group was characterized by high illness in pregnancy, drug use in pregnancy, maternal hazards in pregnancy and unhealthy lifestyle in pregnancy. Specific intervention measures can be carried out for this group, such as pregnancy health education, reducing illness in pregnancy, careful use of drugs, promoting healthy lifestyles, and avoiding unhealthy exposures in pregnancy.

The low-risk cluster (71.46 %) had a prevalence of birth defects of 0.52 %, within an overall lower level of risk factors except for nutrition and folic acid deficiency. Our results were consistent with previous research in Shanxi, which further confirms the importance of nutrition and folic acid use [27].

In our study, annual net income per capita was significantly associated with birth defects. Higher income families were less likely to have birth defects, which is consistent with previous study findings [28]. It is expected that higher income families can afford high nutrition food and good living condition compared to lower income families. Such difference in life style is particular striking according to income level in China. Meanwhile, place of residence (urban/rural) did not show statistical significance for birth defects. Since the subjects in rural and township in our study were living in a socially and environmentally homogeneous geographical area, we do not expect this to be significant for birth defects. As most mothers in the study were junior high school graduates (72.87 %), we also do not expect to see significant difference in birth defects across different maternal education levels.

The population-based retrospective epidemiological survey covered all likely risk factors for birth defects in six counties of Shanxi Province from 2006 to 2008. This was a large sample that reflected the global distribution of birth defects in Shanxi Province. Using a combination of a total risk factor score from an accumulated series of risk factors, and LCCA-extracted synergistic effects of multiple risk factor clusters for dimensionality reduction, our study covered overall maternal exposure factors. Furthermore, as a probability-based method, LCCA has an advantage over distance-based cluster analysis, for example, probability of assignment to *k* latent clusters for each mother [20] and lower misclassifications compared with the *k*-means [29]. Our analysis classified mothers into mutually exclusive risk clusters for birth defects, meaning we could screen the group at high-risk for birth defects in an area with a high-risk of birth defects.

However, our study had some limitations. First, the population-based data obtained was only a preliminary design for risk factors of birth defects [13]. Some exposure classifications such as tobacco smoking and alcohol intake need to be further quantified. Also, as the study was conducted in a heavy coal mining area in China, the pollution characteristics need to be taken account of in the designed questionnaires. A ‘control’ area from other low pollution provinces should be considered to eliminate the confounding effect caused by coal mining. Second, due to the cross-sectional design of the study, cause-effect conclusions cannot be made and the results should be interpreted with caution. Third, our data only included live births occurring in 2006–2008, and excluded stillbirths before 28 weeks [13], which may have accounted for a significant proportion of birth defects. We also did not differentiate between preterm birth and full-term birth in our study, which would be meaningful for detecting maternal exposure risk factors. Further research should work to identify genes controlling risk of birth defects in the context of genotype–phenotype associations.

## Conclusion

Our results show synergistic effects based on overall maternal exposures and screening of expectant mothers at high-risk of birth defects in a high-risk area. Mothers in our sample were divided into three distinct groups with different risks of having offspring with birth defects: a high-risk cluster, a moderate-risk cluster, and a low-risk cluster. This will inform the provision of targeted interventions for women of reproductive age in areas with a high risk of birth defects. Additionally, as there is an increase in available electronic medical chart data for women of reproductive age, screening high-risk groups based on overall maternal exposures with a cluster-based approach may be an important direction for future study and targeted interventions, therefore contributing to birth defects control.

## Abbreviations

AIC: akaike information criterion
AOR: adjusted odds ratio
BIC: bayesian information criterion
CAIC: consistent Akaike information criterion
LCCA: latent class cluster analysis
PFPC: Population and Family Planning Commission
